# Unveiling the shadows: obstacles, consequences, and challenges of information opacity in healthcare systems

**DOI:** 10.1186/s13010-025-00170-6

**Published:** 2025-04-14

**Authors:** Majid Alizadeh, Nazila Azizi, Samireh Mahdavi, Fouad Baghlani

**Affiliations:** 1 Student Research Committee, School of Health Management and Information Sciences, Shiraz University of Medical Sciences, Shiraz, Islamic Republic of Iran; 2 Abadan University of Medical Sciences, Abadan, Islamic Republic of Iran

**Keywords:** Information transparency, Health system, Consequences, Challenges

## Abstract

**Introduction:**

Information transparency in healthcare systems is critical for ensuring public trust, enhancing service quality, and reducing costs. However, many countries face significant challenges concerning information opacity, which leads to inequality, discrimination, and increased risks for patients and healthcare providers. This study aims to explore the obstacles, consequences, and challenges of information opacity in healthcare systems, along with proposing solutions for improvement.

**Method:**

This review synthesized findings from scientific literature, including articles, reports, and governmental sources, to investigate how the lack of information transparency affects healthcare performance and public trust. A comprehensive search was conducted across major databases such as PubMed, Scopus, and Google Scholar, utilizing relevant keywords. Selection criteria focused on the relevance, quality, and timeliness of the sources, leading to a critical analysis of the extracted data through thematic synthesis.

**Findings:**

The study identifies several key consequences of information opacity, including a decrease in public trust, reduced service quality, increased corruption, and heightened healthcare costs. The findings align with existing literature that highlights the importance of transparency for effective decision-making and accountability in healthcare systems. Furthermore, obstacles to achieving transparency were identified, such as difficulties in accessing necessary information, privacy concerns, commercial interests, and the need for systemic reforms in healthcare financing.

**Discussion:**

The implications of this study underscore the necessity for clear policies and procedures regarding information dissemination in healthcare. The proposed framework for improving transparency includes establishing robust communication channels, enhancing public access to information, fostering a culture of accountability, and leveraging emerging technologies like blockchain and artificial intelligence. Addressing these challenges is essential for building trust and improving healthcare outcomes.

**Conclusion:**

Enhancing information transparency within healthcare systems is vital for improving public trust and service quality. This study provides a foundational framework for policymakers to implement necessary changes, promoting a more equitable and efficient healthcare environment. Future research should focus on evaluating the effectiveness of these proposed measures in diverse healthcare contexts, particularly by integrating theoretical frameworks such as stakeholder theory and institutional theory.

## Introduction

Health services are the basic need of all people and all governments are responsible for them. The health system is a system that maintains the health of individuals and communities. This system includes many different aspects, such as disease prevention and treatment, health care, nutrition, physical fitness, and mental health. Many factors are involved in the success or failure of the health system in achieving the desired goals. Among these factors are national policy-making, financial resources management, human capital empowerment, improving the level of health culture, technology development, inter-sectorial cooperation, and information transparency. Information transparency in the health system means easy and timely access to information related to the quality, cost, and performance of health services. Information transparency can help increase public trust, improve efficiency, and reduce health costs [1, 11, 30]. However, in many countries, the lack of transparency of information in the health system is a serious problem that causes patients, providers, and health organizations to experience inequality, and discrimination and be exposed to multiple risks. Transparency of costs, prices, quality, and effectiveness of medical services and products has been identified as a key tool to reduce costs and improve outcomes. Researchers consider the prerequisite for achieving transparency in the health field to be a comprehensive transformation in the structural mechanisms of society, i.e. the external environment of medical structures, and at the same time providing a suitable platform within the organizations providing health services. However, achieving transparency in health care can be challenging due to obstacles such as difficulty in obtaining information that should be made public and the need for fundamental changes in the way hospital care is paid for [16, 25, 36]. Despite these challenges, transparency is an essential feature of open and democratic societies and has the potential to significantly improve consumers'ability to participate in choosing where to receive care [10].

Currently, there exists a significant research gap regarding the broad implications and challenges associated with information opacity in healthcare systems. While previous studies have predominantly focused on specific aspects of this issue, such as limited access to information or its impact on public trust, a holistic and comprehensive perspective encompassing the various factors related to this topic has yet to be fully developed. In this article, we aim to address this gap by presenting a thorough examination of the barriers, consequences, and challenges of information opacity in healthcare systems. Our goal is to provide a new framework that not only deepens understanding of the subject but also offers practical solutions for enhancing transparency.

This paper is structured into several key sections. The methodology section outlines the approach used for data collection and analysis. The findings section identifies the critical consequences of information opacity and highlights existing obstacles. In the discussion section, we explore the link between our findings and proposed improvements, while also analyzing the role of emerging tools such as artificial intelligence and blockchain in fostering greater transparency. This structure enables us to make a unique contribution to the existing literature and address important questions that have remained unanswered thus far.

By adopting this approach, we hope to advance the discourse on transparency in healthcare systems and provide actionable insights for policymakers, practitioners, and researchers alike.

## Method

The objective of this review research is to examine the absence of information transparency in the health system, its consequences, barriers, and challenges. This research was formulated and implemented with the objective to respond to the question: To what extent is the absence of information transparency in the health system impacting service providers'effectiveness, the quality of services provided, patient satisfaction, and public trust?

In order to address the research question, relevant scientific literature concerning the subject matter was sought, reviewed, evaluated, synthesized, and cross-checked. Articles, books, reports, as well as websites belonging to governmental and non-governmental organizations, form the category of scientific literature. Various scientific databases were accessed in looking for appropriate sources. Some of the databases accessed were PubMed, Scopus, Web of Science, and Google Scholar.The keywords"Information transparency,""Health system,""Consequences,""Challenges,"and their Persian equivalents were combined using Boolean operators (AND, OR, NOT) to ensure comprehensive coverage of the literature.

In the next step, among the searched articles, those related to the research question of this study and of suitable scientific quality were selected. For this, criteria such as year of publication, type of source, research method, and results were applied, and irrelevant, outdated, weak, or duplicate sources were excluded. Specifically, only studies published between 2010 and 2024 were included to ensure relevance and timeliness.

In the subsequent phase, the chosen sources were analyzed critically and significant information was drawn out. Similarities and dissimilarities among the sources were determined, and based on the data extracted, relationships, trends, and ambiguous points of the topic of research were examined. In order to resolve the research problem, the information drawn from the articles was examined closely, and the authors could finally develop an extensive and independent perspective of the topic. A thematic synthesis approach was employed to organize the findings into meaningful categories, ensuring a systematic and structured analysis.

## Previous research

Houri eh Faisalabad et al. (2018) in research titled"Effect of organizational transparency on administrative corruption with mutual effects of organizational trust in medical centers of Tehran municipality", means organizational transparency as the means of information disclosure. Based on this definition, organizational transparency requires a broad combination of appropriate attitudes and characteristics of organizational culture. Also, organizational transparency can be seen as a broad concept that implies free access, decision-making, and freedom of information. Providing the information needed by the consumer to make an informed choice will lead to increased transparency in performance evaluation. Also, transparency increases competition, improves organizational trust, and improves the quality of decision-making. In this study, researchers identified three types of transparency: informational transparency, participatory transparency, and accountability transparency, which complement each other and are analytically distinct from each other [15].

Yuri Timofeev et al., in a systematic review study entitled"The impact of transparency limitations on the efficiency of the Russian health care system", have investigated transparency indicators in the world's health systems. According to the purpose of this study, the authors define transparency as the availability and access to information for the public. In particular, this information refers to the activities of institutions of the health care system, such as medical organizations, health authorities, federal funds, and compulsory medical insurance and insurance companies. Transparency includes (a) transparency of conditions for providing medical care to the population, (b) transparency of institutions and laws related to the allocation of resources in the health care system, and (c) transparency of health care results. In this study, transparency in the Russian health system has been proposed as an important tool for improving efficiency. The authors state that existing organizational constraints in the Russian healthcare system create serious obstacles to achieving acceptable levels of transparency. After examining the health system in several countries, the authors have concluded that the lack of a clear purpose for providing information is one of the common weaknesses of most health systems. Also, there is no consensus on who is the main audience, be it patients, government institutions, or other stakeholders. According to the authors, these basic issues require convincing answers because the way of presenting information depends on the correct understanding of the audience [40].

Sonorous Safian, Ph.D. in Political Science, Harvard University; and Wallace J. Hopp, Ph.D., University of Michigan (2019) in a study titled"The Role of Quality Transparency in Health Care: Challenges and Potential Solutions"which aims to investigate challenges and potential solutions to increase quality transparency in the health system and the effects of quality transparency on decisions. Patients and healthcare providers claim that quality transparency in the health system can improve treatment outcomes, but to achieve this goal, the information must be generally useful and presented to the appropriate target population. Also, this study shows that despite the significant effect of transparency in information on the results of treatment and care, the use of this information by patients is very low. I have found that increasing quality transparency can have a positive effect on the behavior of healthcare providers. For example, this transparency can encourage providers to focus on their strengths or make investments to improve their weaknesses. Do it yourself. In general, this study shows that quality transparency can be an effective tool for improving the health system, but achieving this goal requires the correct use of this tool as well as appropriate policy interventions [37]

. David M. Clark et al. [9] in a study entitled"Transparency about mental health service outcomes: Analysis of public data from the IAPT approach"which was conducted to investigate transparency about mental health service outcomes globally, showed that transparency about mental health services outcomes can lead to improvements in mental health services worldwide. By providing information about the clinical outcomes of mental health services, patients can see what services local health and treatment organizations provide and what results they achieve. Also, this transparency allows managers and employees to compare their services with other similar service providers and learn from each other's experiences and solutions. Transparency can also help health organizations to improve their mental health services and perform better [9].

Ding Chanian (2020) in a study entitled"Deadly lack of transparency of information in public health emergencies: lessons from the outbreak of COVID- 19 in China"aims to investigate the lack of transparency of information in public health crises and learn from the outbreak of COVID- 19. 19 has been done in China states that the four main factors responsible for the lack of information transparency in the spread of COVID- 19 in China are: weak disease control and prevention centers, the priority of political concerns over social cohesion, and national coordination over public health, Very strict control of the public media and deficiencies in the public health crisis information system in the face of new and emerging infectious diseases.

The author believes that improving the transparency of information in public health crises requires attention to four basic lessons: strengthening disease control and prevention centers, prioritizing public health over political concerns, promoting freedom of expression and public opinions, and improving health crisis information systems in exposure to new and emerging infectious diseases [8].

Han Abram et al. (2022), in a study entitled"The effect of price transparency and competition on hospital costs", have obtained contradictory results about the effect of price transparency on health costs. The authors of this study state that hospitals in states that have implemented price transparency policies have higher operating costs than states that have not implemented this policy. Also, non-profit hospitals have higher operating costs than for-profit hospitals. In this article, the authors conclude that information transparency in the health system can be an effective tool to reduce hospital costs. They believe that disseminating public hospital performance information can improve healthcare delivery and help consumers make the best healthcare decisions. Also, transparency can stimulate competition and ultimately lead to cost reduction through cost control, quality improvement, and efficiency improvement. However, the authors also point out that transparency may lead to higher prices for health services and may also increase market concentration and reduce competition. Therefore, they believe that to achieve the desired results, additional policy measures should be taken into consideration and, in addition to transparency, also consider market competition [24].

Zara Moon et al. (2021) in a mixed (qualitative-quantitative) study, entitled"Designing an Organizational Transparency Model with an Organizational Health Promotion Approach at Zahedan University of Medical Sciences"aims to identify and rank the components of organizational transparency with a health promotion approach. An organization has been conducted at Zahedan University of Medical Sciences, and they have identified fourteen dimensions affecting organizational transparency, which in order of importance are: information management, transparency, lack of secrecy, religious and spiritual teachings, organizational control, trust and monitoring ability. organizational accountability, human resource management, culture building, administrative automation, organizational Whistleblowing, participation, information technology, and accountability. According to the results of this research, the researchers recommend that senior managers use the most important and efficient tools of transparency, i.e. new communication and information technologies, in all organizational processes to achieve the basic consequences of organizational transparency [29].

Ojjaara Brahmani et al.(2022) in a descriptive-analytical study entitled"Investigation of the relationship between transparency and organizational commitment with the mediating role of organizational trust among hospital employees of Tehran University of Medical Sciences; 1400"aims to analyze the correlation between the relationships of transparency, trust and commitment organization has been done from the employees'point of view, they state that the dimensions of organizational transparency have a direct and positive effect on the dimensions of organizational commitment, also the authors advise managers to identify the hospitals'publishable information by creating a correct understanding of transparency, awareness of priorities and provide the basis for transparency measures, because increasing organizational transparency expands the spirit of cooperation and leads to the improvement of employees'trust and ultimately increases their organizational commitment [35].

In a comparative study conducted by Hossein Bouzarjamehri et al. (2019) entitled"Comparative study of the transparency of the performance of hospitals in eight countries", the formation of different systems for the dissemination of transparent information in hospitals and the successful experiences of different countries in this field have been investigated. Conflicts of interest and legal and ethical issues related to information transparency in the health system were also examined. This study showed that the transparency of the performance of hospitals in different countries is different according to the health system, social and private insurance, private for-profit and non-profit organizations, and government organizations. In this study, the authors have pointed out the challenges and consequences of the lack of transparency in the health system. Some of these challenges and consequences are lack of access to information: lack of transparency can lead to a decrease in public access to information related to the performance of hospitals. This issue can decrease public trust and increase the dissatisfaction of patients and society. Decreasing the quality of services: lack of transparency can lead to a decrease in the quality of services in hospitals. Without access to information related to the performance of hospitals, the possibility of evaluating and improving the quality of services is reduced. Increase in corruption: Lack of transparency can be the basis for corruption in the health system. The existence of opaque information and lack of transparency can provide a good opportunity for corruption and financial and administrative violations. Decrease in public trust: Lack of transparency can cause a decrease in public trust in the health system and hospitals. Without access to information about the performance of hospitals, people cannot properly evaluate and lose their trust in the health system [6].

Lack of transparency in decision-making: Lack of transparency can cause a lack of transparency in decisions related to the health system. This issue can lead to unfair and ineffective decisions in health system improvement policies and programs. The authors conclude that transparency in the performance of hospitals and health services can lead to the improvement of service quality, increase in public trust, reduction of corruption, and better decisions in policies and transformation programs in the health system. Also, the authors recommend that health policymakers in Iran implement appropriate programs and policies to promote transparency in Iran's health system by taking advantage of international experiences and considering the challenges and consequences related to transparency [6]. Foreshore Depasan et al. (2020) in a study entitled"Prevention of administrative corruption in the health system"stated that depriving people of the necessary information causes corruption. Transparency through publishing reports and monitoring compliance of organizations with laws and ethical rules can be effective. Also, anti-corruption programs should include financing and necessary structural reforms. The transparency of information ensures that health service providers do not act in such a way as to raise expectations and provide low-level services in practice [12].

Fatemeh Johari (2014) in a study entitled"Analysis of the issue of clarifying medical errors, analysis of the state of Iran's medical institution"says,"Nowadays, transparency has become a social demand and the public demand for transparency has gone beyond political and economic institutions and extended to medical institutions. It has been found"in this newsletter, the researcher specifically examines the issue of clarifying medical errors and concludes that important measures have been taken in Iran in the field of clarifying medical errors, but due to structural obstacles, there is a long way to achieve full transparency in this field. is ahead He considers the prerequisite for achieving transparency to be a multilateral transformation in the structural mechanisms of society, that is, the external environment of medical structures, and at the same time providing a suitable platform within this institution [14].

Ebrahim Jafari Pouyan et al. [33] in a descriptive-analytical study titled"Hospital managers'desire for transparency of information and related indicators in the health system: consequences and challenges"aims to investigate the desire of hospital managers for transparency of information and related indicators in three dimensions (structural, process, consequence) and identifying the consequences and challenges related to this issue in the health system has been done to the importance of transparency in performance evaluation and health support, review of experiences related to transparency in other countries, problems and challenges in Iran, communication The willingness of managers has paid. Among the most important results of this study, we can mention the following: hospital managers were most inclined to transparency of information related to structural indicators, managers'inclination to transparency of information related to the outcome dimension of hospitals was less, management level, organizational position and education level with a tendency to Transparency had a significant relationship in its various dimensions, and in general, hospital managers had a relatively high desire for transparency of information for the general public. According to the study, the challenges and consequences of the lack of transparency of information in the health system are a Decrease of public trust: The lack of transparency of information can lead to a decrease of public trust in healthcare organizations and services. People need access to reliable and understandable information to make better decisions about their health. Increase in risks and dangers: The lack of transparency of information can lead to an increase in risks and dangers in the healthcare system. Without access to sufficient information, people cannot properly assess existing risks and problems and avoid them. Decreased accountability: The lack of transparency of information can lead to a decrease in the accountability of organizations and officials to public problems and needs. People need access to appropriate information to evaluate the performance of organizations and provide appropriate feedback. Increasing uncertainty and criticism: The lack of transparency of information can lead to increasing uncertainty and criticism about the performance of healthcare organizations and services. Without access to sufficient information, individuals cannot properly assess whether organizations are functioning fully and optimally. Increase in costs: The lack of transparency of information can lead to an increase in costs in the healthcare system [33].

## Findings

### Consequences of information opacity

The lack of transparency in healthcare systems has profound consequences that affect various stakeholders, including patients, healthcare providers, and the public at large. Below are the key findings categorized under this section:

- Decrease in Public Trust: The absence of accessible and reliable information undermines public confidence in healthcare institutions. Patients who cannot access accurate data about service quality or costs may lose faith in the system.
- Reduced Service Quality: Without transparency, it becomes challenging to evaluate and improve the quality of healthcare services. This leads to suboptimal care delivery and dissatisfaction among patients.
- Increased Corruption: Opaque systems create opportunities for unethical practices, financial mismanagement, and administrative violations. Corruption thrives when there is a lack of accountability and oversight.
- Higher Costs: Information opacity often results in inflated healthcare expenses due to inefficiencies, redundancies, and lack of competition.
- Lack of Transparency in Decision-Making: When critical health-related decisions are made without sufficient input from all stakeholders, they risk being unfair or ineffective, further eroding trust in the system.
- Risks and Dangers: Patients exposed to incomplete or misleading information face higher risks of harm, such as medication errors or delayed diagnoses.
- Decreased Accountability: Without clear mechanisms for monitoring performance, healthcare organizations may fail to meet their responsibilities, leading to mistrust and dissatisfaction.
- Uncertainty and Criticism: A lack of transparent communication fosters skepticism and criticism, hindering collaborative efforts between patients, providers, and policymakers.

For a comprehensive overview of the consequences associated with information opacity in healthcare systems, please refer to Table 1, which categorizes and details the key impacts on stakeholders, including decreased public trust, reduced service quality, and increased corruption, among others.

**Table 1:** Consequences of lack of transparency

Category	Details
Decrease in Public Trust	Loss of confidence in healthcare institutions due to inaccessible data
**Reduced Service Quality**	Challenges in evaluating and improving healthcare services
**Increased Corruption**	Opportunities for unethical practices and financial mismanagement
**Higher Costs**	Inefficiencies and redundancies caused by opaque systems
**Lack of Transparency in Decisions**	Unfair or ineffective decision-making processes
**Risks and Dangers**	Higher likelihood of harm due to incomplete or misleading information
**Decreased Accountability**	Failure to hold organizations accountable for their actions
**Uncertainty and Criticism**	Skepticism and criticism due to lack of open communication

### Barriers to achieving transparency

Despite its importance, achieving transparency in healthcare systems faces several significant barriers. These challenges must be addressed to ensure meaningful progress:

- Difficulty in Obtaining Public Information: Many healthcare organizations struggle to release essential data due to legal constraints, privacy concerns, or organizational resistance
- Complex Payment Systems: Fundamental changes in how hospital care is financed are necessary but difficult to implement, particularly in countries with entrenched bureaucratic structures.
- Privacy Concerns: Protecting patient confidentiality while promoting transparency remains a delicate balance, often complicating efforts to share relevant information.
- Data Protection Laws: Regulations designed to safeguard personal health information can sometimes hinder broader transparency initiatives.
- Commercial Interests: Companies prioritizing profits over openness may resist sharing vital data about treatments, pricing, or outcomes.
- Inequality in Access: Disparities in technological infrastructure and literacy levels prevent equitable access to health information across populations.

For a detailed breakdown of the challenges associated with achieving transparency in healthcare systems, including issues such as legal and organizational barriers, privacy concerns, and systemic inequalities, please refer to Table 2, which categorizes and elaborates on these barriers.

**Table 2:** Barriers to achieving transparency

Category	Details
Difficulty in Access	Legal, technical, or organizational obstacles to releasing public information
**Complex Payment Systems**	Need for systemic reforms in financing models
**Privacy Concerns**	Balancing confidentiality with transparency
**Data Protection Laws**	Regulatory frameworks limiting the dissemination of sensitive health data
**Commercial Interests**	Prioritization of profit over openness by pharmaceutical and insurance companies
**Inequality in Access**	Unequal distribution of resources and skills affecting access to health information

### Proposed solutions and recommendations

To address the identified issues, we propose a comprehensive framework aimed at enhancing transparency in healthcare systems. Each recommendation builds on the insights gained through our thematic analysis of existing literature:

1. Establish Clear Policies and Procedures: Policymakers should develop and enforce robust guidelines mandating the disclosure of service quality, costs, and performance metrics. This includes creating standardized formats for reporting and ensuring compliance across all levels of the healthcare system.
2. Provide Access to Information: Health organizations must ensure easy and equitable access to critical data, including details about medical errors, adverse events, and treatment options. Digital platforms could play a pivotal role in facilitating widespread dissemination.
3. Improve Communication: Effective communication strategies are crucial for bridging gaps between providers and recipients. Clear, concise, and culturally appropriate messaging will enhance understanding and engagement.
4. Strengthen Disease Control and Prevention Centers: Enhancing the capacity of these centers ensures better preparedness during public health crises and promotes timely information sharing.
5. Promote Freedom of Expression and Public Engagement: Encouraging open dialogue and feedback loops empowers citizens to participate actively in shaping healthcare policies.
6. Upgrade Health Crisis Information Systems: Investing in advanced technologies enables real-time updates and accurate tracking of health emergencies, fostering trust and cooperation.

For a detailed overview of the proposed solutions and recommendations aimed at enhancing transparency in healthcare systems, including strategies such as establishing clear policies, improving communication, and upgrading health crisis information systems, please refer to Table 3, which outlines the comprehensive framework for improvement.

**Table 3:** Proposed framework for improvement

Category	Details
Establish Clear Policies and Procedures	Standardize reporting formats and enforce compliance
**Provide Access to Information**	Use digital tools to disseminate data equitably
**Improve Communication**	Tailor messages to diverse audiences for maximum impact
**Strengthen Disease Control Centers**	Build resilient infrastructures for crisis management
**Promote Freedom of Expression**	Create platforms for inclusive discussions and feedback
**Upgrade Health Crisis Systems**	Leverage technology for rapid and precise information sharing

### Critical analysis

Through thematic analysis, we identified recurring patterns and underlying themes in the reviewed literature. While previous studies emphasize the benefits of transparency—such as improved service quality and reduced corruption—they also highlight the complexities involved in implementation. For instance, balancing privacy with openness requires nuanced approaches tailored to specific contexts. Furthermore, addressing commercial interests demands regulatory frameworks that incentivize transparency without stifling innovation.

Our findings suggest that achieving transparency is not merely a technical challenge but also a socio-political one. It necessitates collaboration among multiple stakeholders, including governments, private entities, and civil society. By adopting a holistic approach, healthcare systems can overcome existing barriers and harness the full potential of transparency to deliver better outcomes for all.

## Discussion

In recent decades, the concept of transparency in healthcare systems has emerged as a cornerstone for ensuring public trust, enhancing service quality, and promoting accountability. Transparency involves providing clear, accessible, and understandable information about the performance of healthcare organizations and institutions. This critical concept plays a pivotal role in fostering public confidence, improving decision-making processes, and reducing corruption within healthcare systems [18, 23, 26, 40]. However, despite its acknowledged importance, achieving effective transparency remains fraught with challenges and obstacles.

### Consequences of lack of transparency

The absence of transparency in healthcare systems has far-reaching consequences that affect multiple stakeholders, including patients, providers, and policymakers. Key findings from this study align with existing literature, highlighting several significant outcomes:

- Decreased Access to Information: A lack of transparency restricts public access to vital information regarding hospital performance, leading to diminished public trust and increased dissatisfaction among patients and communities [22, 32].
- Reduced Service Quality: Without transparent data on hospital performance, evaluating and improving service quality becomes challenging, ultimately compromising patient care [4, 21].
- Increased Corruption: Opaque systems create opportunities for unethical practices, financial mismanagement, and administrative violations, undermining the integrity of healthcare delivery [31, 41].
- Erosion of Public Trust: When individuals cannot access reliable information about healthcare services, their confidence in the system diminishes, further exacerbating distrust [7, 17].
- Ineffective Decision-Making: The absence of transparent processes hampers informed decision-making at both organizational and policy levels, resulting in unfair or suboptimal policies [28, 34].

These consequences underscore the urgent need for systemic reforms aimed at enhancing transparency and accountability in healthcare systems.

### Obstacles to achieving transparency

While the benefits of transparency are evident, numerous barriers hinder its implementation. These challenges can be categorized into several key areas:

1. Privacy Concerns: Protecting patient privacy while promoting transparency remains a delicate balance. Some individuals may hesitate to share sensitive health information due to fears of misuse or breach [2, 38].
2. Data Protection Laws: Regulatory frameworks designed to safeguard personal health information sometimes impose restrictions that impede broader transparency efforts [44].
3. Commercial Interests: Companies prioritizing profits over openness may resist sharing comprehensive data about treatments, pricing, or outcomes, thereby perpetuating opacity [42, 43].
4. Inequality in Access: Disparities in technological infrastructure and literacy levels prevent equitable access to health information, undermining inclusivity in decision-making processes [39, 43].
5. Organizational Resistance: Certain organizations may oppose transparency initiatives due to concerns about exposing inefficiencies or negative performance metrics [3, 18].
6. Political Factors: Health information is often politicized, with some stakeholders using it as a tool for power and influence rather than public benefit [20].

Additionally, the complexity of health-related information, lack of transparent institutions, and conflicts of interest further complicate efforts to establish open and accountable systems.

### Integrating emerging technologies and theoretical frameworks

To address these challenges, innovative solutions leveraging emerging technologies such as blockchain, artificial intelligence (AI), and digital patient portals hold promise. Blockchain technology ensures secure and immutable records, fostering trust among stakeholders. AI-powered analytics enable the processing of vast datasets, making complex information more accessible and actionable. Furthermore, integrating theoretical frameworks like stakeholder theory and institutional theory provides valuable insights into aligning transparency initiatives with societal norms and expectations [5, 13, 19, 27].

#### Impact on stakeholders

Enhancing transparency directly impacts various stakeholders within the healthcare ecosystem:

- Patients: Improved access to information empowers patients to make informed decisions about their care.
- Providers: Transparent systems encourage accountability and motivate providers to improve service quality.
- Policymakers: Access to accurate data enables evidence-based policymaking, driving systemic improvements.

### Policy implications

From a policy perspective, fostering transparency requires:

- Establishing robust regulatory frameworks that balance privacy with openness.
- Investing in digital infrastructure to ensure equitable access to health information.
- Encouraging collaboration between public and private sectors to promote transparency initiatives.

### Future research directions

This study identifies several avenues for future research:

- Evaluating the effectiveness of proposed transparency measures across diverse healthcare contexts.
- Investigating the role of cultural and contextual factors in shaping transparency perceptions and practices.
- Exploring innovative approaches to overcoming resistance and fostering a culture of openness within healthcare organizations.

The accomplishment of transparency in healthcare systems is crucial for reclaiming public trust, enhancing the quality of care, and assuring equitable access to services. Despite the presence of considerable challenges, tackling them through strategic interventions, technological innovations, and theoretical frameworks provides a foundation for the development of more transparent and accountable healthcare systems. Policymakers, practitioners, and researchers will need to act together to develop sustainable solutions to foster transparency as an integral value of contemporary healthcare.

For a comprehensive synthesis of the consequences, obstacles, and challenges associated with achieving transparency in healthcare systems, please refer to Table 4, which categorizes and elaborates on these critical aspects, including decreased access to information, privacy concerns, and systemic complexitie.

**Table 4:** Summary of consequences, obstacles, and challenges

Category	Details
Consequences	- Decreased access to information- Reduced service quality- Increased corruption- Erosion of public trust- Ineffective decision-making
Obstacles	- Privacy concerns- Data protection laws- Commercial interests- Inequality in access- Organizational resistance- Political factors
Challenges	- Complexity of information- Lack of transparent institutions- Conflicts of interest- Political influences- Insufficient access to resources

## Conclusion

This review highlights the pervasive issue of information opacity in healthcare systems, emphasizing its detrimental effects on public trust, service quality, and cost efficiency. The lack of transparency not only escalates healthcare costs and fosters corruption but also restricts access to essential information, hindering effective care delivery and patient satisfaction.

Achieving transparency faces significant challenges, including systemic barriers such as limited data accessibility, outdated payment models, and structural deficiencies. These obstacles are exacerbated by privacy concerns, regulatory constraints, organizational resistance, and commercial interests that prioritize profit over openness. Despite these hurdles, the benefits of transparency—improved health outcomes, enhanced resource management, and restored public trust—are compelling reasons for action.

To address these issues, we propose a strategic framework:

1. Establish Clear Policies: Mandate the disclosure of service quality, costs, and outcomes through comprehensive policies.
2. Enhance Information Access: Ensure patients have easy access to critical data, including details on medical errors and adverse events.
3. Improve Communication: Foster clear, comprehensible exchanges between providers and patients.
4. Strengthen Infrastructure: Develop robust frameworks for disease control and crisis management.
5. Promote Public Engagement: Encourage open dialogue and feedback mechanisms.
6. Upgrade Information Systems: Implement advanced tools for real-time, accurate data sharing during crises.

While achieving full transparency requires overcoming complex challenges, its potential to enhance healthcare quality and equity makes it an indispensable goal. Policymakers and stakeholders must prioritize transparency as a foundational pillar of modern healthcare systems, driving progress toward more open, efficient, and trustworthy services globally.

## Data Availability

No datasets were generated or analysed during the current study.
